# Ultrasound-guided genicular nerve radiofrequency ablation: A revised protocol illustrated through high-definition video

**DOI:** 10.1016/j.inpm.2025.100643

**Published:** 2025-10-18

**Authors:** Tomás Caroço, Giovanni Leoni, Daniela Teixeira, Jorge Ribeiro, Bruno Paiva, Jorge Barbosa

**Affiliations:** a Department of Physical Medicine and Rehabilitation, Hospital de Faro, Unidade Local de Saúde do Algarve, Faro, Portugal; b Algarve Biomedical Center Research Institute (ABC-RI), Faro, Portugal; c Department of Physical Medicine and Rehabilitation, Unidade Local de Saúde de Lisboa Ocidental, Lisbon, Portugal; d NOVA Medical School, Faculdade de Ciências Médicas, NMS, FCM, Universidade Nova de Lisboa, Lisbon, Portugal

**Keywords:** Chronic knee pain, Genicular nerve, Ultrasound-guided intervention, Radiofrequency ablation, Interventional pain management, Nerve to vastus intermedius, Educational video, Knee osteoarthritis

## Abstract

**Background:**

Radiofrequency ablation (RFA) of the knee joint may be an effective therapeutic option for the management of chronic knee pain. Accurate anatomical knowledge is essential for optimizing the precision and outcomes of the procedure. The innervation of the knee joint is complex. Ultrasound (US) guidance offers a widely accessible method for targeting sensory nerves. Given the limited availability of high-quality video literature on knee RFA, we developed an educational video illustrating an extensive revised ultrasound-guided protocol.

**Objective:**

To present a video-based description of a revised ultrasound-guided RFA protocol for the knee, targeting specific nerves by anatomical quadrant: superomedial quadrant (Video 1)– medial branch of the NVI, SMGN; inferomedial quadrant (Video 2) – IMGN; superolateral quadrant (Video 3) – lateral branch of the NVI, SLGN; and inferolateral quadrant (Video 4) - RFN.

**Methods:**

Seven patients with advanced knee osteoarthritis, unresponsive to conservative treatment, underwent selective RFA of either the medial or lateral quadrants, based on the distribution of their pain. The procedures were recorded in high definition and annotated to serve educational purposes.

**Conclusions:**

The video-based illustrations presented may enhance procedural clarity and facilitate clinician training. Due to the time consuming and potential for pain associated with the procedure described, targeting all nerves may be not appropriate in every patient. An individualized approach, tailoring the selection of targets based on patient-specific characteristics and localization of pain is likely more optimal.

## Highlights

• Video-based literature on genicular nerve ablation remains limited.
• This article presents a high-resolution, picture-in-picture video protocol for nerve targeting.
• Radiofrequency ablation of the knee is a complex and uncomfortable procedure.
• Radiofrequency ablation of the knee protocol should be tailoredto improve outcomes and reduce discomfort.

## Ethical approval and informed consent

All procedures involving human participants were conducted in accordance with institutional and national ethical standards and with the 1964 Helsinki Declaration and its later amendments. The use of anonymized video recordings for educational and research purposes was approved by the *Comissão de Ética para a Saúde do Hospital de Faro*, under reference number 0.46.25, on March 20, 2025. All participants provided informed consent for the use of anonymized images and video recordings. The privacy rights of all individuals were fully respected and preserved.

## Text version of video audio voiceover

There is no voiceover; all instructions are provided as on-screen text within the video.

## Declaration of competing interest

The authors declare that they have no known competing financial interests or personal relationships that could have appeared to influence the work reported in this paper.

## Appendix A Supplementary data

Multimedia component 1 Video 1: Ultrasound-guided radiofrequency ablation protocol – superomedial quadrant of the knee, targeting the medial branch of the nerve to vastus intermedius (NVI) [1] and the superomedial genicular nerve (SMGN) [1]. Multimedia component 1

Multimedia component 2 Video 2: Ultrasound-guided radiofrequency ablation protocol – inferomedial quadrant of the knee, targeting the inferomedial genicular nerve (IMGN) [1]. Multimedia component 2

Multimedia component 3 Video 3: Ultrasound-guided radiofrequency ablation protocol – superolateral quadrant of the knee, targeting the lateral branch of the NVI [1] and the superolateral genicular nerve (SLGN) [2]. Multimedia component 3

Multimedia component 4 Video 4: Ultrasound-guided radiofrequency ablation protocol – inferolateral quadrant of the knee, targeting the recurrent fibular nerve (RFN) [3]. Multimedia component 4
